# Citizen surveillance for environmental monitoring: combining the efforts of citizen science and crowdsourcing in a quantitative data framework

**DOI:** 10.1186/s40064-016-3583-5

**Published:** 2016-10-28

**Authors:** Marijke Welvaert, Peter Caley

**Affiliations:** 1 Commonwealth Scientific and Industrial Research Organisation, Canberra, Australia; 2 Plant Biosecurity Cooperative Research Centre, Canberra, Australia

**Keywords:** Citizen science, Crowdsourcing, General surveillance, Environmental statistics, Data generation process

## Abstract

Citizen science and crowdsourcing have been emerging as methods to collect data for surveillance and/or monitoring activities. They could be gathered under the overarching term *citizen surveillance*. The discipline, however, still struggles to be widely accepted in the scientific community, mainly because these activities are not embedded in a quantitative framework. This results in an ongoing discussion on how to analyze and make useful inference from these data. When considering the data collection process, we illustrate how citizen surveillance can be classified according to the nature of the underlying observation process measured in two dimensions—the degree of observer reporting intention and the control in observer detection effort. By classifying the observation process in these dimensions we distinguish between crowdsourcing, unstructured citizen science and structured citizen science. This classification helps the determine data processing and statistical treatment of these data for making inference. Using our framework, it is apparent that published studies are overwhelmingly associated with structured citizen science, and there are well developed statistical methods for the resulting data. In contrast, methods for making useful inference from purely crowd-sourced data remain under development, with the challenges of accounting for the unknown observation process considerable. Our quantitative framework for citizen surveillance calls for an integration of citizen science and crowdsourcing and provides a way forward to solve the statistical challenges inherent to citizen-sourced data.

## Background

Over the last decade, citizen science has become popular in many scientific areas. Involving citizens in scientific research has benefits for both parties. Scientists get access to a wealth of data that would not have been attainable by individual researchers, while citizens get the satisfaction of being involved in science and being part of a community project. In addition, crowdsourcing offers another data stream in which the general public collects or processes data that are used for scientific studies. However, citizen science and crowdsourcing seem to live parallel lives. While for citizen science the focus is on citizens doing science (e.g. data collection, conducting experiments, etc.), in crowdsourcing the volunteers contribute resources (e.g. computing power, money, data, etc.) to science.

**Fig. 1:**
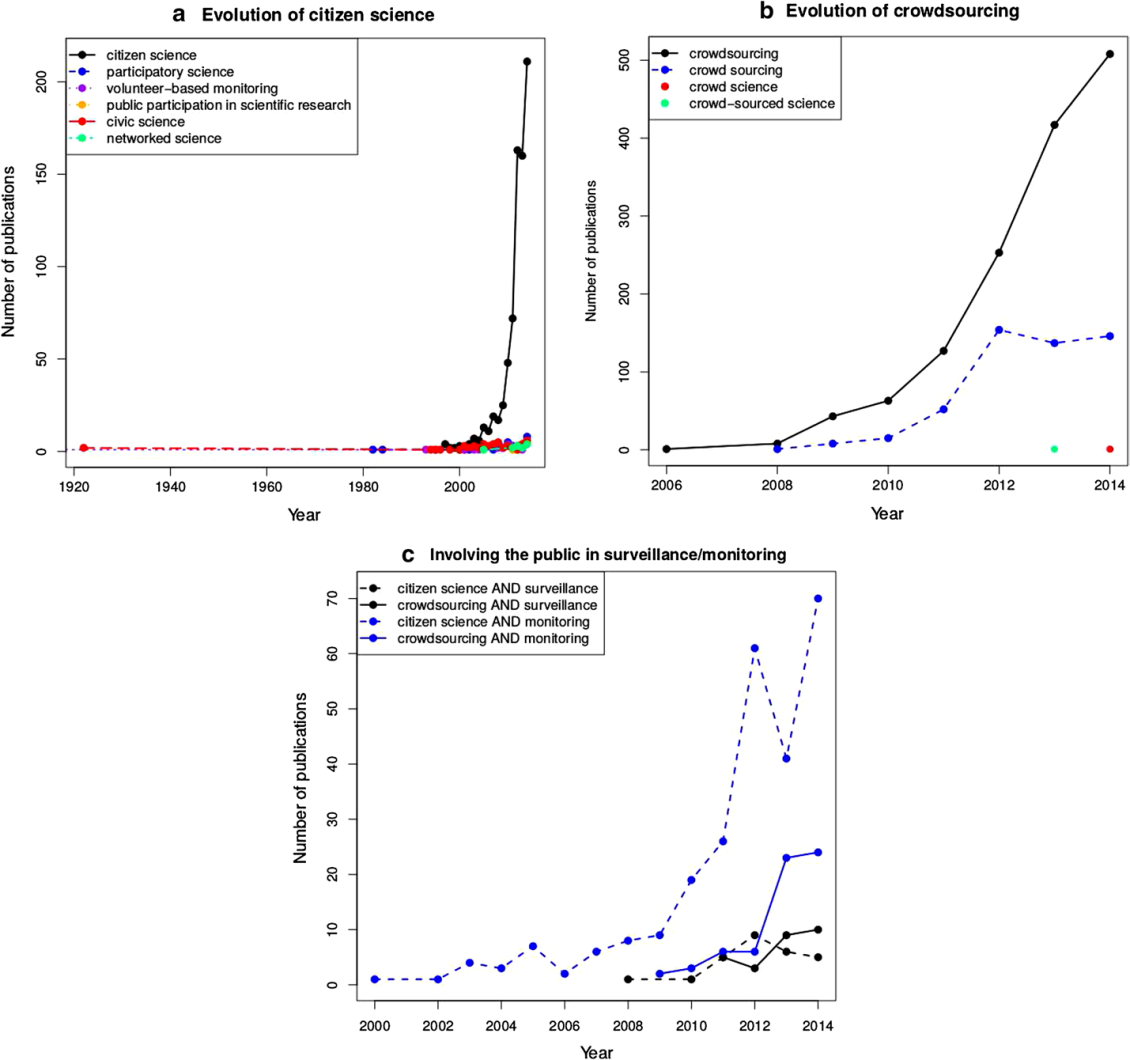
Annual Web of Science publication hits for queries related to **a** citizen science, **b** crowdsourcing, and **c** combining citizen science or crowdsourcing with surveillance or monitoring

Figure 1 demonstrates that both disciplines have attracted increasing interest from the scientific community. Searching for publications relating to citizen science and crowdsourcing using the Web of Science database clearly shows that the number of publications has risen exponentially during the past 5 years. Interestingly, Fig. 1c indicates that citizen science and crowdsourcing has become increasingly popular for monitoring and surveillance activities. Furthermore, according to Roy et al. (2012), many citizen science projects are making use of crowdsourced data. Therefore, it seems natural to consider both disciplines as potential data sources for monitoring and surveillance activities.

In this paper, we propose a quantitative data-focused framework for characterizing citizen surveillance. We define citizen surveillance as *any type of activity conducted by volunteers, recruited or not, that results in monitoring or surveillance data*. Natural application domains for citizen surveillance are environmental surveillance, biodiversity monitoring and biosecurity surveillance, and both citizen science and crowdsourcing are logical subdisciplines of citizen surveillance in this context.

Other reviews (for example Bonney et al. 2009) have mainly focused on how to develop citizen science projects and the benefits in terms of science knowledge and scientific literacy for the communities involved. Although these frameworks include data analysis as an important aspect of the citizen science project, data validation and analysis is still a little studied part of the possibilities of citizen science (Butt et al. 2013). Citizen science, and in this paper we will extend this to citizen surveillance, still struggles to be accepted as a valid scientific method (Bonney et al. 2014) and is often associated with some skepticism with respect to the quality of the data generated. Therefore, not surprisingly, a lot of effort has gone in assessing whether volunteer collected data are of similar quality as data collected by professionals or experts. Recently, quite a number of studies have revealed a strong agreement between volunteer-collected data and empirical or expert-collected data (e.g. Davies et al. 2012; Hoyer et al. 2012; Gollan et al. 2012). However, Snall et al. (2011) warn to be cautious when using citizen science data because these data should be handled with great care. When using citizen science data for environmental surveillance purposes, one needs to be aware of and account for the limitations of those data. For example, Gardiner et al. (2012) demonstrated an overestimation of species richness and diversity values in lady beetles based on citizen science data compared to verified data. This was attributed to under-reporting of common species combined with over-reporting of rare native species (including false positives). Accounting for such findings can clearly improve the analysis of citizen surveillance data.

By focusing on the quantitative aspects of citizen science and crowdsourcing in relation to the reporting and detection process, we hope to provide a way forward to a unified search for statistical solution for citizen-sourced data. We discuss the trade-off between data quality and data quantity in citizen surveillance data, which datasets can be expected from the different types of citizen surveillance and we highlight the analytic challenges which go hand in hand with citizen surveillance data. We hope that by focusing on the quantitative data aspects of citizen surveillance, users will be guided in how to choose an appropriate type of citizen surveillance for their environmental research questions or monitoring goals.

The remainder of this paper is organized as follows: “The emergence of citizen science” section provides a brief overview of the history of citizen-sourced data and presents the results of a extensive literature analysis demonstrating the lack of studies investigating statistical methods for analysing citizen-sourced data. “Citizen surveillance data: a trade-off between data quality and data quantity” section goes into the details of the observation process of citizen surveillance and proposes a new framework that differentiates between different types of citizen surveillance based on common elements in the observation and reporting process. This framework is used to assess the statistical challenges for citizen surveillance. Finally, in “Conclusion” section we provide some recommendations for maximizing the information retrieved from citizen surveillance data and urge for an integrated approach in developing statistical methods for citizen-sourced data.

## The emergence of citizen science

Citizen science may be a rather new term, but in fact its practice dates back several centuries. Before the twentieth century, scientific work was most often conducted by *gentleman scientists* such as Isaac Newton, Abraham Franklin and Charles Darwin. It is only since the mid-twentieth century that science has become almost completely professionalized with scientists employed by universities and research organizations. While this is still mostly the case, over recent decades, several projects have emerged that have successfully engaged volunteers without specific scientific training into posing research questions, and collecting and analyzing data (e.g. Trumbull et al. 2000).

Citizen science has been defined as “the systematic collection and analysis of data; development of technology; testing of natural phenomena; and the dissemination of these activities by researchers on a primarily avocational basis” (OpenScientist 2011). In short, citizen science is commonly known as “public participation in scientific research” (e.g. Hand 2010) and is sometimes referred to as *crowd science*, *crowd-sourced science*, *civic science*, or *networked science*. The term “citizen science” has become commonly used by both scientists (see Fig. 1) and the community from 2009 onward (see Bonney et al. 2009 for a definition of the fiels). It is believed that citizen science is a cost-effective means to provide solutions to research questions, because, in general the data is cheaper than that gathered by trained professionals, while similar in quality and quantity after intelligent analyzing and processing (Barbier et al. 2012). In particular, citizen science can be helpful when researchers attempt to answer questions on a large spatial or temporal scale (Bonney et al. 2009) that cannot be sampled otherwise due to limited funding.

Citizen science projects are increasing in popularity (see Theobald et al. 2015; See et al. 2016; Fig. 1). In order to assess whether this is also the case in the scientific literature, we conducted a literature search using the Web Of Science database, indexing scientific publications going back as far as 1864, and the query “citizen science”. The search resulted in 379 publication hits (results as of December 5, 2013). After inspection, 33 of these (8.7%) were considered irrelevant because they were not related to citizen science as we defined above, another 5 publications (1.3%) were disregarded because they had no assigned author and therefore their geographical source could not be traced. After removing these results, 342 publications remained that all discussed citizen science, although in many different forms.

A content-based analysis of the database revealed that the most common publication type related to citizen science were applications (i.e. 45.6%). This included reporting on the analysis and interpretation of citizen science data (e.g. Wilson et al. 2013); the development of educational projects using citizen science was discussed (e.g. Zoellick et al. 2012); the implementation of a new citizen science project was described (e.g. Arvanitidis et al. 2011); or reporting the discovery of new species as a result of citizen science (e.g. Collins et al. 2011; Cohen et al. 2011).

Only 5.8% of the publications discuss specific analysis challenges and methods to analyze citizen science data, while another 12.2% present studies that tried to validate the citizen science data by, for example, comparing the quality of the data gathered by volunteers on the one hand and by expert professionals on the other. This illustrates that data validation and analysis is indeed understudied. Interestingly, more than half of the citizen science publications found here were published in an environmental related field, namely *Environmental Sciences & Ecology*, *Biodiversity & Conservation*, *Zoology*, and *Entomology*. These fields carry a tradition of large monitoring surveys and projects aimed to assist the conservation of species and it is obvious that citizen science is already well embedded. However, we will demonstrate that not all types of citizen surveillance will be the optimal choice to gather data for conservation monitoring.

## Citizen surveillance data: a trade-off between data quality and data quantity

Citizen science projects can be classified into different types depending on several factors. For example, Roy et al. (2012) derived different clusters of citizen science projects based on how they scored on two dimensions. The first dimension accounted for the *degree of mass participation* (i.e. mass participation projects versus local monitoring projects), while the second dimension was interpreted as the *degree of investment* (i.e. investment of project managers, investment of background information and supporting material, and the time-investment of the participants). In this paper, we follow a similar thinking process in terms of classifying different types of citizen surveillance into a two-dimensional system. However, we specifically focus on the quantitative characteristics of the citizen-sourced data, because they are more relevant with respect to choosing an analysis method.

### Structuring citizen surveillance based on observation and reporting

Citizen-sourced surveillance/monitoring data, in particular from the natural environment, will depend jointly on the processes of species detection/observation and reporting. Factors relating to detection and reporting can be used to characterize the data observation process, with consequences for data preprocessing, appropriate statistical analysis and ultimately the amount of useful information that can be retrieved from the data. To our knowledge, thus far this distinction between the two processes has not been made. Here we argue, differentiating between these processes is crucial for making inference from these data.

**Fig. 2:**
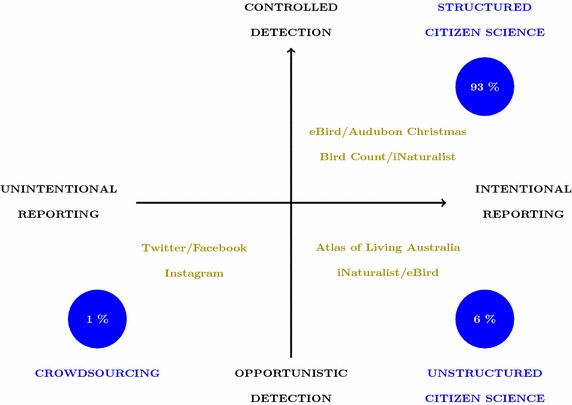
Schematic representation of the citizen surveillance framework. The different types of citizen surveillance are distinguished within a two-dimensional system representing the detection process and the reporting process. Examples of well known data sources for the different types are represented in *green*. The *blue circles* represent the number of publications for each type in our literature review database

Figure 2 illustrates how three broad types of citizen surveillance can be situated in the quadrants of a detection–reporting axis system. On the detection axis, a distinction is made between controlled and opportunistic detections. Controlled detection implies that the time and place of the detection is part of a sampling design, while opportunistic detections are side-products from other day-to-day activities (e.g. day hiking) and therefore there is less control on where and when these detections occur. On the reporting axis, we differentiate between unintentional and intentional reporting. This dimension mainly refers to the platform to which the citizen detections are being reported. Intentional reporting is typically made on a dedicated citizen science platform (e.g. website), while an unintentional reporting is made on a medium that is not directly related to citizen science (e.g. social media).

**Fig. 3:**
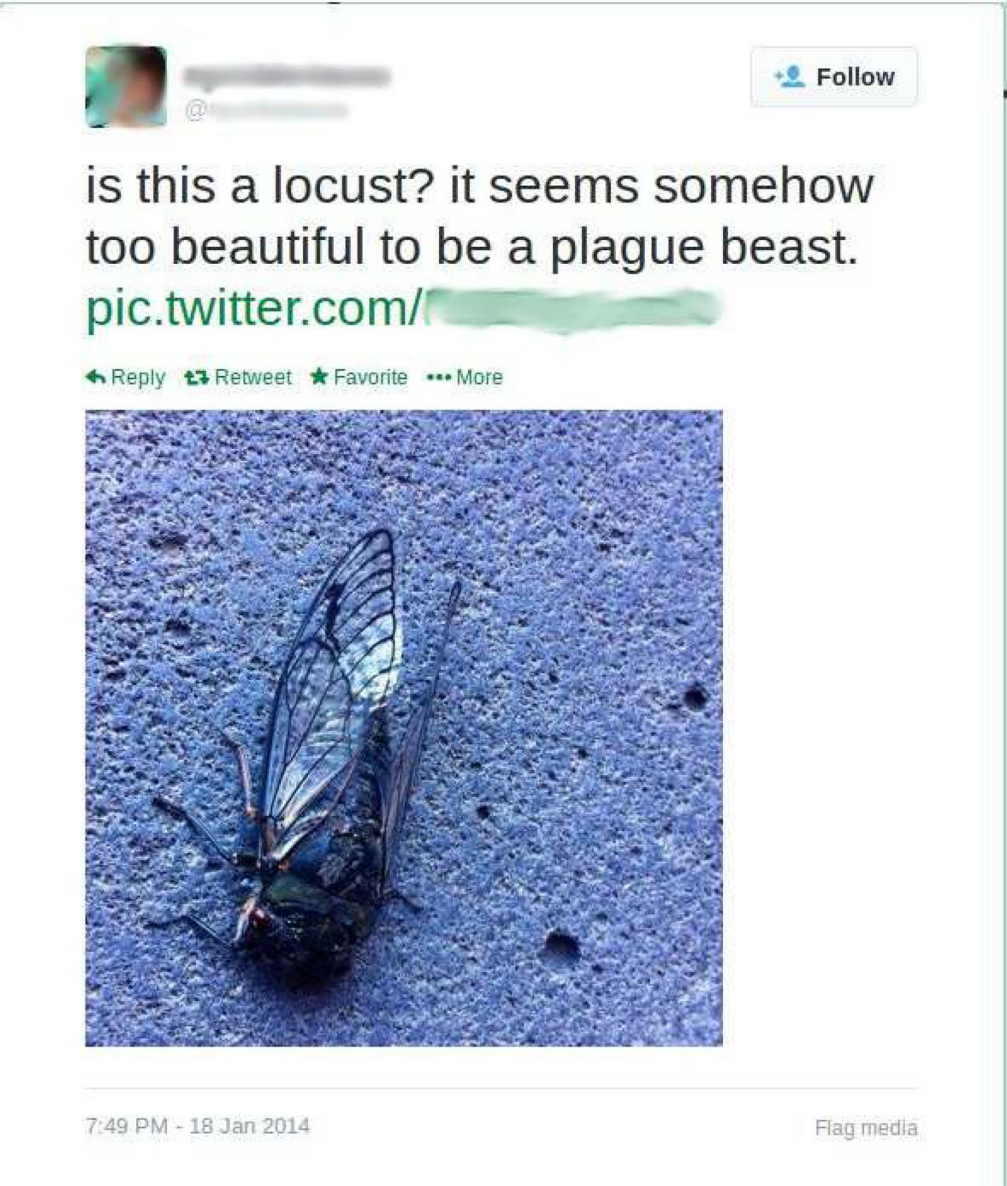
Example of crowdsourcing from Twitter. The photographic material makes it possible to correct the misidentification in the tweet. In this case it is a cicada, unrelated to locusts

Within this two-dimension system, we note that three types of citizen science can be differentiated: (1) Structured citizen science projects are projects that are typically organized by a scientist (or using scientific principles) and in which strong control is exercised on the observation and reporting process. These projects typically offer some form of training to the volunteers, which are purposefully recruited, and provide questionnaires and checklists. In some cases even, an experimental design is adopted. (2) In unstructured citizen science, the reporting is still done on purpose, but the observation process is opportunistic. Observations are typically spontaneous and a result of trips planned or undertaken independently with respect to the species reported. There is typically no information available on the time spent in the field, nor information on other species recorded on the same visit to a given site. This type of citizen science typically needs to be enabled by a web-platform on which volunteers can report their detections. (3) A last category is crowdsourcing. This term captures information that is observed in an opportunistic way and reported unknowingly. For example, Tweets reporting something, sometimes supported by photographic material (see Fig. 3). Crowdsourcing is often not regarded as citizen science because the volunteers are not necessarily involved in the scientific research (Miller-Rushing et al. 2012). However, within the context of our framework, this data stream could be a natural part of citizen surveillance, keeping in mind that it will have its own statistical challenges and does not necessarily provide the same benefits to the community (e.g. scientific literacy).

The literature analysis described in “The emergence of citizen science” section showed that the scientific literature is dominated by the first type, structured citizen science (Fig. 1). The close involvement of scientists in these types of projects is the most probable explanation for this over-representation. As mentioned before, crowdsourcing is often not regarded as citizen science, and therefore, is most likely under-represented in the database presented in “The emergence of citizen science” section.

Figure 3 also illustrates one of the data quality issues inherent to citizen science (i.e. misidentification), whereby a tweet contains both “plague” and “locust”—seemingly referring to an outbreaking species that may cause extensive crop damage. The subject of the tweet (as revealed by a photo) is a harmless species. One of the benefits of the framework shown in Fig. 2 is that we can clearly identify a trade-off along the diagonal axis between data quality and data quantity. Table 1 summarizes some of the advantages and disadvantages for the different types of citizen science.

Data quality issues have been heavily investigated in the assessment of crowd-sourced data for mapping purposes (Antoniou and Skopeliti 2015; See et al. 2013; Jokar Arsanjani et al. 2015). While a lot of the issues related to this field are inherently embedded in citizen surveillance for environmental monitoring, we do believe that the data quality problems go even further due to the differences in the data generating process. For example, disaster detection like earthquakes based on georeferenced information relies on the fact that the public will be reporting about the disaster using media that are georeferenced, e.g. Twitter. This works quite well because people immediately recognise the disaster (the observation) and it affects them in such manner that they feel inclined to Tweet about it (the reporting). However, in environmental monitoring, both of these processes are challenged. The observation will be subject to bias and false positives because some species, especially rare/exotic species and/or small species, are hard to detect and it can be very difficult to differentiate between the species to the untrained eye. In addition, the mere observation of a species will not always trigger a reporting response from the observer, unless they have a vested interest in reporting that species or are highly motivated to do so.

Because of its underlying scientific principles, structured citizen science has the major advantage that there is relative control over the observation process. This might be the reason why this type is so present in the scientific literature. On the other hand, unstructured citizen science and crowdsourcing are subject to many uncontrolled processes, most of them unknown. The challenge in analyzing the data from these types of citizen science will be accounting for unknown observation processes and coming up with ways to incorporate the increased uncertainty. In addition, the differences in data type will trigger different analysis strategies. While structured citizen science most likely will provide counts or presence-absence data, unstructured citizen science and crowdsourcing will mainly provide presence-only data. This further complicates the inference that can be made from these data and is amongst statisticians a topic of ongoing debate (Royle et al. 2012; Hastie and Fithian 2013).

Another important aspect is data quantity. Typically, the more data that are available, the more likely it is that uncertainty in the data can be accounted for (i.e. partition signal from noise). However, in citizen science data this is not necessarily true. Data quantity is linked to the signal-to-noise ratio. For example, crowdsourcing provides huge datasets but the signal of interest in these data is only a small fraction and can be totally buried in the noise. Analysis of these data will require a lot of preprocessing and will be extremely hard to validate. There might be less data from structured citizen science, but the signal-to-noise ratio will be a lot higher, meaning that the information in the data is more easily accessible.

Temporal and spatial coverage are important characteristics for environmental surveillance in particular. Rather than having data for one location in a specific time period, environmental monitoring and biodiversity monitoring can benefit immensely from data that cover a lot of space and that are observed frequently. Because structured citizen science is mostly, but not always, an organized activity that brings together a lot of people at the same time at the same place, it is expected to have less spatial and temporal coverage compared to unstructured citizen science and crowdsourcing. The joint effort in these types of citizen surveillance will most likely cover more ground and time, but are also prone to spatial and temporal biases due to opportunistic sampling (see “Challenges” section).

**Table 1:** Evaluation of some typical characteristics of citizen surveillance data

	Type of citizen science		
	Structured	Unstructured	Crowdsourcing
Control over observation process	${+}{+}$	${-}$	${-}{-}$
Data quantity	${-}/{+}$	${-}/{+}$	${+}$
Signal-to-noise ratio	${+}{+}$	${-}/{+}$	${-}{-}$
Temporal coverage	${-}/{+}$	${+}$	${+}$
Spatial coverage	${-}/{+}$	${+}$	${+}$

Choosing a type of citizen surveillance will typically be a trade-off between data quality and data quantity

Symbol legend: ${+}{+}$ Good; ${+}$ Moderate; ${-}/{+}$ Variable; ${-}$ Low; ${-}{-}$ Bad

### Challenges

#### Sampling design and analysis

We have already demonstrated that citizen surveillance can vary from highly structured to completely unstructured. Structured surveillance can be found in avian monitoring, which has a long tradition of citizen science and, not surprisingly, has also been the origin of improved sampling designs for large monitoring studies (e.g. Heezik and Seddon 2012; MacLeod et al. 2012). In contrast, for unstructured citizen surveillance, a statistically sound sampling design is normally lacking and most data are collected based on convenience sampling (Boakes et al. 2010). This means that sampling locations are often near roads or other easily accessible locations, and sampling is most likely on weekends and during daylight hours. In addition, both inter- and intra-observer sampling effort is highly variable and will result in heterogeneity of detection probabilities. The previously mentioned analysis methods, while potentially useful for structured citizen science projects, do not apply to the unstructured part of citizen surveillance. In these cases, induced heterogeneity will have to be accounted for in the analysis of the data.

Developing validated methods for correcting sampling bias for such data is an active area of research in the species distribution modelling field (e.g. Fithian et al. 2015), including the use of high-quality data from planned surveys to leverage information from presence-only data (Dorazio 2014). Other bias correction mechanisms have a logical basis (e.g. using the density of roads as a covariate in the case of citizen-sighted koalas), though are applied without knowing how well the correction is performing (see Sequeira et al. 2014).

**Fig. 4:**
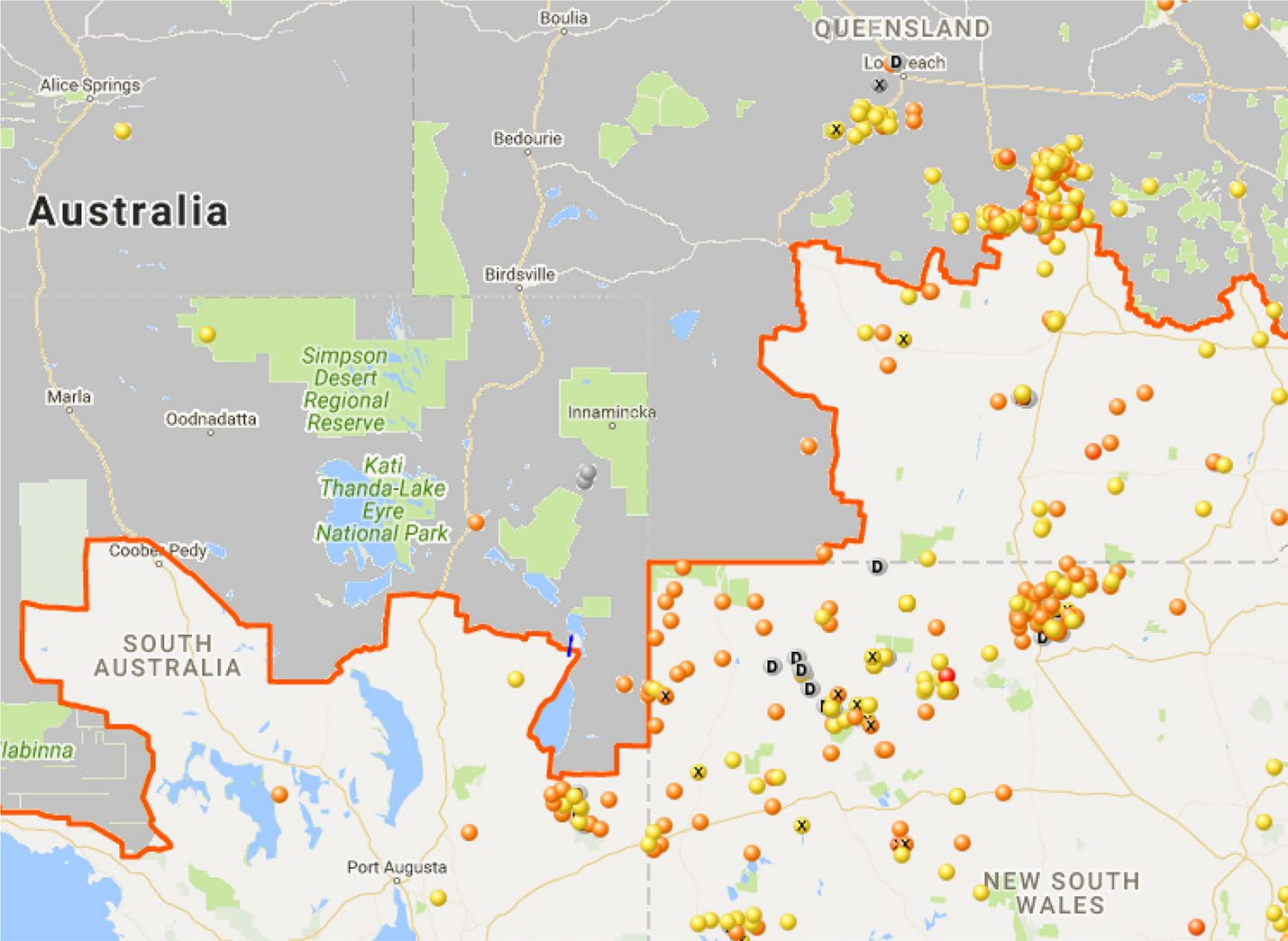
Sightings and evidence of activity of wild dogs (dingoes, domestic dogs and their hybrids) from inland southeastern Australia uploaded to Wild Dog Scan (https://www.feralscan.org.au/wilddogscan/; Accessed 12 September, 2016). The *orange line* marks the position of the dingo barrier fence that separates areas of high dingo density to the north of the fence (*shaded grey*) from areas of very low dingo density to the south within the sheep pastoral zone

In some situations, however, no amount of correction can remedy observer reporting (c.f. sampling) bias inherent in unstructured citizen science. For example, Wild Dog Scan (https://www.feralscan.org.au/wilddogscan/) enables landholders in Australia to report sightings of dingoes (*Canis lupus dingo*) and their hybrids. Such sightings are of particular interest to the sheep pastoral industry, as dingoes may cause severe economic losses. The resulting dingo sightings map is dominated by sightings within the sheep pastoral zone, despite dingoes being rare in this region as a result of intensive control, in comparison with areas to the north outside the dingo barrier fence, where dingoes are comparatively abundant (Fig. 4). Recognising that these data are unstructured should alert anyone using them to research the observation process before undertaking any analysis—failure to do so in this case would result in the incorrect inference that dingo abundance is highest inside the dingo fence. In this situation the sightings probability being somewhat perversely inversely related to abundance is driven by sightings being of much greater economic importance to graziers inside the dingo fence than out. This is not to say that the reporting App and the data it generates aren’t of use to users within the dingo fence.

In the case of crowdsourcing involving unintentional reporting in conjunction with opportunistic detection, there has been little development of validated methodology for retrieving stable surveillance information these type of data.

#### Observer effects

Because citizen surveillance often relies so heavily on inexperienced volunteers, observer effects due to heterogeneity in skills, age, background, etc. can be non-trivial and should not be ignored. Not only do they affect species detectability, they also will influence the adopted sampling design and ultimately the error probability. In the case of citizen science, probably most forms of bias in the data can be traced back to the observers, either through their observing skills, or their choice of sampling locations and time. However, reducing these effects through, for example, standardization may not be sufficient to eliminate the heterogeneity as there are other variables that will influence species detectability (Yoccoz et al. 2001). Sauer et al. (1994) illustrate that long-term trends in observer quality can substantially bias estimates of population trend.

In general, it is known that novices and non-experts are more likely to over-report rare species that are easy to detect (Sullivan et al. 2009). On the other hand, self-assessed experts may suffer more from overconfidence (Larrick et al. 2007), making them more prone to false-positive errors (Moore and Healy 2008).

However, these observer effects can be either controlled through training with structured citizen science, or accounted for provided information is available on, for example, survey effort, experience, etc.

#### A way forward

Merely pointing out the challenges arising from using citizen surveillance data is obviously not enough. When making inference from those data, sampling bias and observer effects will have to be accounted for. In our proposed framework (Fig. 2) we made a clear distinction between the observation and reporting process. Most of the challenges discussed above were looked at in the light of the observation process simply because little is known on how the reporting process is affected. We can speculate that there will be similarities in the types of bias arising in both processes, but there might also be differences. From a statistical perspective, we would like to account for as much bias as possible to ensure valid inference and it would be particularly useful if we could even distinguish between the different sources of potential bias.

Given the rise of web-platforms enabling unstructured citizen science, we believe that effort should be targeted to developing statistical methods for analysing these data. Because of their less controlled observation and reporting process, compared to structured citizen science, sampling and reporting bias will play a major role (as illustrated by the Wild Dog example discussed earlier) and will need to be accounted for. However, this will only be possible, if information around sampling effort or knowledge around what drives the reporting is available. Only then statisticians can try to develop methods that incorporate such information to improve inference from unstructured citizen science.

## Conclusion

In this paper, we advocate an integrated view on citizen surveillance that combines the existing efforts of citizen science and crowdsourcing. Based on the characteristics of the observation process, both disciplines can be naturally combined in one quantitative framework that allows for an integrated approach to solve the statistical challenges inherent to citizen-sourced data.

See et al. (2016) also present a two-dimensional framework to distinguish types of crowdsourced geographic information based on who collects the data (government agencies versus general public) and the contribution characteristic of the data (active versus passive). Their framework no doubt contributes to structuring the thinking around crowdsourced geographic information, however, it does not cover off on the specifics of the data collection process as practiced in citizen surveillance for environmental monitoring. In particular, no distinction is being made between the actual observation process of a species versus the reporting process of a species. In this review, we have highlighted the importance of this distinction as knowledge of those processes, or the absence of such knowledge, will have to inform the inference methods applied to these data.

Our framework distinguished between three major types of citizen surveillance:

1. *Structured citizen science*
  Currently the most published form of citizen surveillance and also the type that aligns most with the current understanding of citizen science. The use of scientific principles, the potential for training of participants and the overall high level of involvement of the volunteers creates a data stream that has the potential to provide high-quality data that are in a format (e.g. counts) that can be analyzed with readily available tools. However, because of its reliance on funding, the temporal and spatial scope can be limited.
2. *Unstructured citizen science*
  Enabled by online platforms like iNaturalist (http://www.inaturalist.org), unstructured citizen science provides access to an audience that is intrinsically motivated to report observations. These datasets are more prone to sampling and observer bias, but additional data fields in the recording process can allow for the collection of meta-data that can be incorporated in the analysis process. However, statistical techniques to make useful inference of these data are not readily available at this point in time and more effort will have to be targeted to the validation of these techniqes if we want to rely more on unstrictured citizen science for environmental monitoring.
3. *Crowdsourcing*
  Crowdsourcing offers an untapped resource of surveillance data with contributions to, for example, social media increasing constantly. Successful examples include, but are not limited too, the tracking of human disease (Culotta 2010) and resource allocation after disasters (Zhu et al. 2011). However, analyzing these data for environmental surveillance purposes will be a huge challenge because the information of interest can be buried amongst numerous irrelevant posts. Methods to reliably retrieve this information are still being developed. In addition, both unstructured citizen science and crowdsourcing will most commonly provide presence-only data and the analysis of these data is still an active topic of discussion amongst statisticians (Royle et al. 2012; Hastie and Fithian 2013).

This framework that classifies citizen surveillance data is a way forward to achieve the full potential of those data. Indeed, such classification models are a necessity because they point to the importance of describing the sampling process and the observation process (Bird et al. 2014). This information is crucial during the analysis process as it can be used to improve statistical models for citizen surveillance data.

Initiatives like eBird (http://www.ebird.org) make it possible to collect environmental data world-wide. While current efforts go mainly in engaging volunteers, data storage and data sharing, there is still a major role for statisticians to guide citizen surveillance projects with better ways to collect their data and to improve statistical models for citizen surveillance data.
